# The correlations between serum bone biomarkers and those related to metabolic and hormonal profile, low-grade inflammation and redox balance, in lean and overweight PCOS adolescent girls

**DOI:** 10.3389/fnut.2025.1477992

**Published:** 2025-07-07

**Authors:** Małgorzata Mizgier, Veronica Sansoni, Barbara Więckowska, Grażyna Jarząbek-Bielecka, Dorota Formanowicz, Witold Kędzia, Giuseppe Banfi, Giovanni Lombardi

**Affiliations:** ^1^Department of Sports Dietetics, Chair of Dietetics, Faculty of Health Sciences, Poznan University of Physical Education, Poznan, Poland; ^2^Laboratory of Experimental Biochemistry and Advanced Diagnostics I.R.C.C.S. Ospedale Galeazzi-Sant’Ambrogio, Milano, Italy; ^3^Department of Computer Science and Statistics, Poznan University of Medical Sciences, Poznan, Poland; ^4^Center of General Sexology and Sexology of Developmental Age, Division of Gynecology, Department of Gynecology, Poznan University of Medical Sciences, Poznan, Poland; ^5^Department of Medical Chemistry and Laboratory Medicine, Poznan University of Medical Sciences, Poznan, Poland; ^6^Vita-Salute San Raffaele University, Milano, Italy; ^7^Department of Athletics, Strength and Conditioning, Poznań University of Physical Education, Poznań, Poland

**Keywords:** polycystic ovary syndrome, adolescent girls, obesity, overweight, bone health

## Abstract

**Introduction:**

It has been proven that polycystic ovary syndrome (PCOS) is associated with reduced bone mineral density (BMD) and impaired bone metabolism. However, to the best of our knowledge, neither the relationship between indices of bone turnover in adolescent girls was examined, nor were lean and overweight PCOS young females compared in this regard, which were the aims of our study.

**Methods:**

Thirty-nine PCOS subjects, aged 14–18 years, were assigned to one of the two groups: Ov/Ob (overweight/obese group, *n* = 14) and lean (non-overweight/non-obese group, *n* = 25). Fasting blood samples were collected to assess bone turnover, inflammation, oxidative stress, and hormonal markers. Basic anthropometric and biochemical data were also obtained.

**Results:**

In Ov/Ob young females, concentrations of bone turnover markers, GlaOC, GluOC, and CTX-I (selective bone resorption marker), were lower than in lean PCOSs. However, this difference was statistically significant only for GlaOC. The serum activity of bone alkaline phosphatase (BAP), a bone formation index, tended to be higher in the Ov/Ob than in lean PCOS patients, although not significantly. Additionally, we observed an inverse association between low-grade inflammation, oxidative stress, androgen levels (total testosterone and/or DHEA-S), and BAP and/or GlaOC in both lean and Ov/Ob groups, together with a positive association between Total Antioxidant Capacity (TAC) and BAP. Moreover, fasting glucose, insulin, and HOMA-IR positively correlated with GluOC and BAP in lean girls.

**Discussion:**

Our outcomes suggest a potential negative interaction between bone markers and immune-hormonal abnormalities featuring lean and Ov/Ob adolescent PCOS girls. Moreover, these findings suggest a positive interaction between bone metabolism and total antioxidant capacity, and insulin and glucose management exists in the body. Although these findings require further investigation, all possible preventive measures should be taken to lower inflammation, oxidative stress, and androgen levels, also keeping bone well-being/homeostasis in mind.

## Introduction

Polycystic ovary syndrome (PCOS) is the most common endocrinopathy during the reproductive age in women and adolescent girls and is also one of the most frequent hyperandrogenic syndromes. In women, hyperandrogenism is a state of increased production of androgens, which manifests with menstrual disorders, lack of ovulation, hirsutism, and, often, seborrhea and acne. Women with hyperandrogenism show a significantly higher incidence of infertility, metabolic dysfunctions, excessive body and fat mass, atherosclerosis, insulin resistance, and diabetes (1–3).

It should be highlighted that most women and girls with PCOS are either overweight or obese. Obese adolescents with PCOS have a more severe metabolic and hormonal profile than lean adolescents with PCOS. They experience an increased production of androgens due to the increased conversion of hormones in adipose tissue or stimulated by hyperinsulinemia and their excessive production in the ovaries and adrenal glands. A reduced concentration of sex hormone-binding globulin (SHBG) may also cause the pool of free hormones to be higher than in lean girls. Therefore, obese girls are at higher risk of menstrual disorders, hirsutism, and developing PCOS (4–6). In particular, visceral obesity and excess adipose tissue may exacerbate insulin resistance (IR), dyslipidemia, and hormonal disorders (6–10).

Bone metabolism is known to be deregulated in PCOS (11). It has been proven that abnormalities in bone metabolism that translate into reduced bone mineral density (BMD) and deregulated circulating levels of indexes of bone metabolism, such as bone alkaline phosphatase (BAP), C-terminal cross-linked telopeptide of type I collagen (CTX-I), osteocalcin (OC) are associate with metabolic syndrome in overweight PCOS adolescents (12). OC exists in various forms with different degrees of carboxylation, with the limit forms being the fully carboxylated (GlaOC) and the fully uncarboxylated (GluOC). Both are believed to be involved differently in regulating insulin secretion and sensitivity. They may be related to metabolic and hormonal abnormalities in PCOS (8, 9), although their role in regulating energy metabolism in humans has not been defined. From a turnover point of view, GlaOC may be regarded as a marker of bone formation. In contrast, Glu-OC, which is supposed to be a mediator of energy metabolism, may be considered a resorption marker (13).

Further, PCOS is also associated with chronic low-grade inflammation (LGI) status and a deregulated redox balance that is associated with increased oxidative stress (OS) (14–16). Both states predispose to poor bone health in PCOS women. Indeed, it has been proved that in PCOS adult females, the strongest negative predictor of radial bone strength-strain index (SSI) (measured by peripheral quantitative computed tomography) is inflammation, measured by CRP/albumin ratio (17).

In previous studies conducted by our group, we observed that, in young PCOS patients, the mean and median concentrations of markers related to oxidative stress and inflammation were increased, whereas the total antioxidant capacity (TAC) was decreased in overweight and obese patients when compared to their lean counterparts (18). We have also revealed that the hormonal and metabolic profiles are impaired in young PCOS compared to healthy controls (19) but also in overweight and obese PCOS patients compared to the matched lean PCOS group (10, 20). On the other hand, it turned out that the androgen level alteration, LGI, oxidative stress, and hyperinsulinemia, which are expressions of PCOS pathophysiology, may affect bone cell metabolism in PCOS women aged 23–47 years (11, 21–25).

Because of the more severe PCOS phenotype in overweight/obese girls related to hormonal and metabolic features and related to inflammation and OS, we aimed to investigate whether there is any difference in bone turnover, assessed through the measurement of bone turnover markers (BAP, CTX-I, GlaOC, and GluOC), between overweight/obese and lean PCOS adolescent girls. Further, we aimed to assess the association between bone turnover markers and metabolic, hormonal, inflammatory, and redox changes in overweight/obese and lean young PCOS females. The knowledge about the role of PCOS pathophysiology in bone remodeling in women and adolescent girls is of great interest since this represents the age of intensive growth and development, and bone growth has an outstanding role during this phase.

## Materials and methods

### Study characteristics and participants

#### Participants

The study’s inclusion was based on the 2003 Rotterdam PCOS diagnostic criteria (clinical and/or biochemical hyperandrogenism, oligo−/amenorrhea, polycystic ovary image in an ultrasound examination) (1).

Thirty-nine PCOS subjects, aged 14–18 years, were assigned to one of the two groups: Ov/Ob (overweight/obese group, *n* = 14) and lean (non-overweight/non-obese group, *n* = 25). Diagnosis and classification of overweight and obesity were based on body mass index (BMI), according to the World Health Organization (WHO) for children aged 5–19 years (26).

The girls were matched for age and pubertal developmental criteria. Only girls who achieved 4 or 5 points on the Tanner scale were included (27).

Exclusion criteria included any systemic chronic diseases, e.g., thyroid dysfunctions, diabetes mellitus, congenital adrenal hyperplasia, Cushing’s syndrome, hyperprolactinemia suggestive of pituitary adenoma and androgen-secreting tumors, as well as chronic medications, hormone-replacement therapies or antibiotic treatments in the last 3 months, vitamins or supplements (involved in bone and carbohydrate metabolism), alcohol consumption and smoking. The eligibility criteria of the research have previously been described in detail (10, 18–20).

#### Ethics and dissemination

This study was conducted in accordance with the Helsinki Declaration and has been approved by the Bioethics Committee of the Poznań University of Medical Sciences (approval no. 553/18, add. 161/20, add. 416/22). All participants and their parents or legal guardians were informed about the procedures and the associated benefits and risks and signed an informed consent before the enrolment.

This study was registered retrospectively at ClinicalTrials.gov (accessed on 4 February 2021) (ClinicalTrials.gov Identifier: NCT04738409) since registration was not required when study enrolment started.

#### Data availability

##### Biochemical analyses and medical evaluation

During a 2-day hospital stay, the gynaecologist specialized in adolescent gynaecology (G J-B) investigated any symptoms of hormonal disturbances, based on the patient’s history, physical examination, blood tests, and transabdominal ultrasound examination.

After overnight fasting, blood samples (2 × 7.5 mL) were taken in the early follicular phase (3rd–5th day). Six hundred μL-aliquots were prepared to be analysed immediately after collection or storage. Blood analysis procedures are described in detail in (28).

Hormones, including total testosterone, androstenedione, dehydroepiandrosterone-sulphate (DHEA-S), together with sex hormone-binding globulin (SHBG) and metabolic indexes (i.e., fasting glucose and insulin), were measured immediately after collection. Biochemical parameters were analysed by electrochemiluminescent immunoassay (ECLIA) (Elecsys, Roche Diagnostics GmbH, Mannheim, Germany) at the central laboratory of the Gynecology and Obstetrics Hospital. The homeostatic model assessment of insulin resistance (HOMA-IR) was calculated using the following formula: HOMA-IR = (fasting plasma glucose [mg/dL] × fasting plasma insulin [μU/mL])/405 (18).

Selected immune and inflammatory parameters [tumor necrosis factor-alpha (TNF*α*), C-reactive protein (CRP), interleukins 1 and 6 (IL-1 and IL-6)], redox balance profile [total antioxidant capacity (TAC), malondialdehyde (MDA)], and androstenedione concentrations were assayed at the Department of Medical Chemistry and Laboratory Medicine (Poznań University of Medical Sciences). All analytes were assayed in duplicate using commercially available ELISA kits: IL-1, IL-6, TNF-α, and MDA were assayed with the commercial ELISA test from SunRed, China. CRP was measured using a commercial ELISA test from DRG, Germany. TAC was assayed by a colorimetric (photometric) microplate assay from Omnidiagnostica Forschungs GmbH, Austria, and the results are expressed as mmol/L Trolox equivalent. All the tests were read on a microplate reader TECAN (Switzerland) with the software Magellan (Switzerland) (27).

Bone turnover markers (CTX-I, BAP, GlaOC, GluOC) were investigated at the Laboratory of Experimental Biochemistry (IRCCS Ospedale Galeazzi-Sant’ Ambrogio, in Milan, Italy). Samples were stored and transported at −80° C for these assays until analysed.

##### Anthropometric assessment and body composition measurements

Anthropometric measurements included height and weight, while BMI was calculated. Diagnosis and classification of overweight and obesity were based on BMI, according to the World Health Organization (WHO) for children aged 5–19 years (28). Body composition was evaluated with a Bioelectrical Impedance Analyzer (BIA; Tanita MC780). Fat mass (FM) measurements were expressed as percentages (%) and kilograms (kg). Anthropometrical assessment and body composition measurement procedures are detailed in (10, 18, 29, 30).

### Statistical analysis

This study used statistical tests appropriate to the type of variables analysed and their distribution. For comparisons between two independent groups and for quantitative variables with a normal distribution, the unpaired t-test (t-st) was used. However, and the Cochran-Cox (C-C) correction was used in situations of different group variances. The Mann–Whitney (M-W) test was used if the distribution was not normal. The existence of correlations between variables was also tested. For quantitative variables with a normal distribution, the statistical significance of the Pearson coefficient was tested. In the absence of normality of the distribution, the statistical significance of the Spearman coefficient was used. The normality of data distribution was tested using the Shapiro–Wilk test, while equality of variances was assessed using the Fisher-Snecor test. The data are described consistently using means, standard deviations, medians, and quartiles. Results were interpreted as statistically significant based on *p*-values less than 0.05. PQStat 1.8.6 software was used for calculations.

Due to the limitations related to the fact that only girls who achieved 4 or 5 points on the Tanner scale might be included in the study, in determining the necessary sample size, we assumed that we would only be looking for large effect sizes. A large effect size when comparing two independent groups, according to Cochen’s interpretation, is the value of the difference in means concerning the standard difference in measures that exceeds 0.8. Assuming a standard level of statistical significance and power of 80%, and assuming that one of the groups was approximately twice as large as the other, we obtained a necessary sample size of more than 19 people in the minority group. As a result, the differences in group size turned out to be smaller, and thus, the minority group (Ov/Ob) in this study was 14 people, and the majority group (Lean) was 25. In addition to detecting large differences when comparing independent groups, such a group size also ensures the detection of strong correlations.

**Figure 1:**
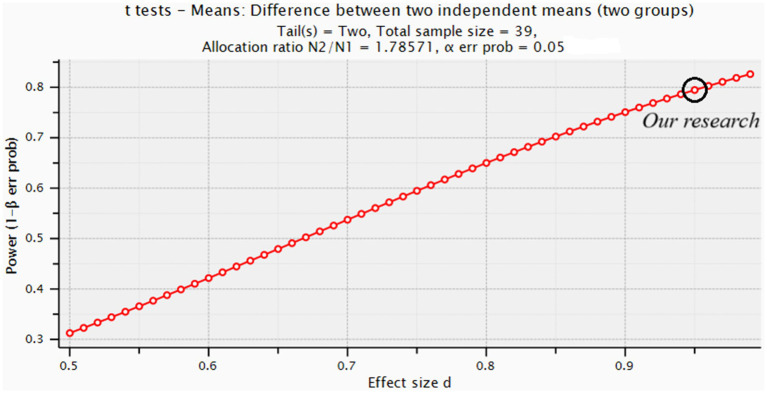
Shows the achieved statistical power for our current sample size *n* = 39 (14 vs. 25) across a range of effect sizes (from small to very large).

To assess the statistical power of our study, we conducted a post hoc power analysis based on the achieved effect sizes and sample sizes (14 vs. 25). As shown in Figure 1, our study was adequately powered to detect very large effect sizes (Cohen’s d ≥ 0.95), which were observed for several key outcomes. Cohen’s d = 0.95, means the mean difference between groups is 0.95 standard deviations. When such large effects were present in our data, they achieved statistical significance. This means that any effects that did not reach statistical significance in our study were smaller than the threshold we could reliably detect.

## Results

### Basic characteristics

The participants’ basic characteristics, including anthropometric indexes, and body analysis, are presented in Table 1. Compared to lean girls, Ov/Ob girls were characterized by significantly higher BMI (30.89 ± 3.94 vs. 20.04 ± 2.18; *p* < 0.0001, respectively), weight (86.54 ± 10.65 vs. 54.98 ± 6.94; *p* < 0.0001, respectively), fat mass (FM) expressed as a percentage (FM%) (*p* < 0.0001) and kilograms (FMkg) (*p* < 0.0001), waist circumference (WC) (*p* < 0.0001), and waist-to-hip ratio (WHR) (*p* = 0.0003). There were no differences in age, height, and hip circumference (Table 1).

**Table 1:** Basic characteristics of the participants, including anthropometric indexes, and body analysis.

Parameters	Ob/Ov (*n* = 14)	Lean (*n* = 25)	*p* value	Test
BMI			***<0.0001***	(C-C)
Mean ± SD	30.89 ± 3.94	20.04 ± 2.18		
Median [Q1; Q3]	30.72 [28.09; 33.09]	19.57 [18.49; 21.85]		
Age (years)			0.12	(M-W)
Mean ± SD	15.5 ± 1.7	16.46 ± 1.02		
Median [Q1; Q3]	15.5 [14; 17]	16 [16; 17]		
Height (m)			0.37	(t-st)
Mean ± SD	1.67 ± 0.05	1.66 ± 0.06		
Median [Q1; Q3]	1.68 [1.65; 1.7]	1.66 [1.6; 1.71]		
Weight (kg)			***<0.0001***	(t-st)
Mean ± SD	86.54 ± 10.65	54.98 ± 6.94		
Median [Q1; Q3]	85.75 [80.63; 92.58]	53.65 [50.88; 56.95]		
FM %			***<0.0001***	(t-st)
Mean ± SD	34.44 ± 7.41	20.83 ± 5.46		
Median [Q1; Q3]	34.9 [28.5; 40.05]	19.35 [16.68; 25.73]		
FM Kg			***<0.0001***	(M-W)
Mean ± SD	21.19 ± 10.35	8.02 ± 4.68		
Median [Q1; Q3]	17.85 [14.58; 24.88]	6.8 [4.78; 9.68]		
WC [cm]			***<0.0001***	(C-C)
Mean ± SD	93.64 ± 9.73	71 ± 5.82		
Median [Q1; Q3]	91.5 [89.25; 97.75]	69.5 [67.75; 74.5]		
HIP [cm]			0.06	(M-W)
Mean ± SD	107.57 ± 13.21	99.42 ± 13.14		
Median [Q1; Q3]	111.5 [93; 116.25]	96 [90.5; 107.25]		
WHR			***0.0003***	(t-st)
Mean ± SD	0.88 ± 0.13	0.73 ± 0.11		
Median [Q1; Q3]	0.84 [0.79; 0.96]	0.72 [0.69; 0.78]		

BMI, body mass index; FM-fat mass; WC, Weight circumference; HIP, Hip circumference; WHR, Weight to hip ratio.

The analysis was conducted using an unpaired *t*-test (t-st) or Mann–Whitney test (M-W).

All the *p*-values marked in bold and italics were statistically significant.

### Inflammatory, hormonal, metabolic markers, and oxidative stress measures

Mean serum concentrations of the inflammatory markers IL-1, IL-6, TNFα, and CRP, as well as of the oxidative stress marker MDA, tended to be higher in the Ov/Ob group than in the lean group, although they did not reach the statistical significance. TAC tended to be higher in the lean group than in the Ov/Ob group (Table 2).

**Table 2:** Serum concentration of hormonal and immune-metabolic parameters in overweight and obese, and lean females.

Parameters	Ob/Ov (*n* = 14)	Lean (*n* = 25)	*p* value	Test
IL-1 [pg/mL]			0.25	(M-W)
Mean ± SD	36.1 ± 26.92	28.02 ± 16.35		
Median [Q1; Q3]	26.87 [23.27; 33.45]	25.46 [17.18; 30.58]		
IL-6 [ng/L]			0.34	(M-W)
Mean ± SD	44.5 ± 40.6	32.73 ± 18.52		
Median [Q1; Q3]	28.56 [25.15; 39.53]	27.33 [21.77; 35.75]		
TNF-alfa [ng/L]			0.41	(M-W)
Mean ± SD	129.34 ± 122.22	95.31 ± 77.4		
Median [Q1; Q3]	87.09 [62.99; 113.88]	77.72 [56.79; 103.63]		
CRP [mg/L]			0.42	(M-W)
Mean ± SD	1.48 ± 1.83	1.06 ± 1.43		
Median [Q1; Q3]	0.83 [0.66; 1.08]	0.66 [0.4; 1.17]		
TAC [mmol/L]			0.64	(t-st)
Mean ± SD	1.02 ± 0.22	1.06 ± 0.2		
Median [Q1; Q3]	1.02 [0.89; 1.17]	1.02 [0.9; 1.17]		
MDA [nmol/mL]			0,42	(M-W)
Mean ± SD	12.44 ± 12.33	9.44 ± 8.39		
Median [Q1; Q3]	7.5 [6.35; 10.26]	6.9 [5.07; 9.54]		
Fasting glucose [mg/dL]			***0.04***	(t-st)
Mean ± SD	90.65 ± 7.89	86.24 ± 4.96		
Median [Q1; Q3]	89.55 [86.2; 95.88]	86.5 [81.58; 88.6]		
Fasting insulin			***0.02***	(M-W)
Mean ± SD	20.58 ± 10.18	13 ± 6.34		
Median [Q1; Q3]	19.56 [12.14; 30.26]	12 [8.62; 15.03]		
HOMA-IR			***0.009***	(M-W)
Mean ± SD	5.1 ± 2.85	2.81 ± 1.5		
Median [Q1; Q3]	4.56 [2.75; 6.96]	2.46 [1.79; 3.22]		
Total T [nmol/L]			***0.007***	(M-W)
Mean ± SD	2.14 ± 0.44	1.63 ± 0.65		
Median [Q1; Q3]	2.21 [1.83; 2.54]	1.56 [1.32; 1.86]		
Free testosteron			***0.0004***	(M-W)
Mean ± SD	11.39 ± 4.3	5.78 ± 3.98		
Median [Q1; Q3]	11.95 [7.43; 14.5]	4.35 [3.8; 7.78]		
Androstenedione [ng/mL]			***0.0004***	(M-W)
Mean ± SD	7.28 ± 3.66	2.98 ± 2.61		
Median [Q1; Q3]	8.2 [3.75; 9.9]	2.02 [1.59; 4.03]		
DHEA-S			0.08	(M-W)
Mean ± SD	8.11 ± 2.94	6.66 ± 2.43		
Median [Q1; Q3]	7.85 [6.38; 9.18]	6.32 [5.28; 7.18]		
SHBG [nmol/L]			***0.002***	(M-W)
Mean ± SD	37.94 ± 19.75	75.21 ± 41.69		
Median [Q1; Q3]	30.79 [21.55; 47.12]	59.1 [46.97; 101.01]		

IL-1, Interleukin-1; IL-6, Interleukin; TNFα, Tumor Necrosis Factor α; CRP, C-reactive protein; TAC, total antioxidant capacity; MDA, malondialdehyde; HOMA-IR, Homeostatic Model Assessment of Insulin Resistance; Total T, total testosterone; Free T, free testosterone; DHEA-S, dehydroepiandrosterone; SHBG, sex hormone-binding globulin.

The analysis was conducted using an unpaired *t*-test (t-st) or Mann–Whitney test (M-W).

All the *p*-values marked in bold and italics were statistically significant.

Regarding the metabolic markers, fasting glucose (*p* = 0.04), fasting insulin (*p* = 0.02), and HOMA-IR (*p* = 0.009) were significantly higher in Ob/Ov PCOS than in their lean counterparts (Table 2).

Further, the Ov/Ob group differs unfavorably and significantly from the Lean group according to the serum concentration of hormonal parameters: total T (*p* = 0.007), free T (*p* = 0.0004), androstenedione (*p* = 0.0004), and SHBG (*p* = 0.002). There are no significant differences between the groups in serum concentrations of DHEA-S, but in the Ov/Ob group, the mean and median values tended to be higher than in the lean group (Table 2).

### Bone biomarkers

In overweight and obese females, the mean and median concentrations of bone formation markers Gla-OC, Glu-OC, and bone resorption marker CTX-I were slightly lower in comparison to the matched group, but a significant difference was only observed for Gla-OC. Whereas serum concentration of Bone Alkaline Phosphatase (BAP) - a marker of bone formation was slightly higher in the Ov/Ob group than in the group of lean girls, the difference was not significant (Table 3).

**Table 3:** Serum concentrations of bone formation markers in overweight and obese, and lean females.

Parameters	Ob/Ov (*n* = 14)	Lean (*n* = 25)	*p* value	Test
BAP [U/L]			0.25	(M-W)
Mean ± SD	29.12 ± 14.95	22.8 ± 9.35		
Median [Q1; Q3]	23.01 [18.86; 32.73]	20.88 [16.25; 29.33]		
CTX-I [ng/mL]			0.58	(t-st)
Mean ± SD	0.69 ± 0.28	0.74 ± 0.25		
Median [Q1; Q3]	0.65 [0.54; 0.79]	0.73 [0.57; 0.88]		
GlaOC [ng/mL]			***0.002***	(M-W)
Mean ± SD	6.36 ± 2.79	9.19 ± 3.72		
Median [Q1; Q3]	5.01 [4.51; 7.34]	8.32 [6.87; 10.93]		
GluOC [ng/mL]			0.67	(M-W)
Mean ± SD	13.26 ± 3.02	13.35 ± 2.99		
Median [Q1; Q3]	12.75 [11.42; 15.54]	14.18 [11.77; 15.32]		

The analysis was conducted using an unpaired *t*-test (t-st) or Mann–Whitney test (M-W).

All the *p*-values marked in bold and italics were statistically significant.

### Correlations among bone biomarkers and metabolic and hormonal parameters, and markers of inflammation and oxidative stress

BAP was significantly correlated with markers of inflammation and redox balance. Namely, inverse correlations were observed between BAP and CRP (*r* = −0.63; *p* = 0.01) in Ov/Ob group; in the lean group of patients, BAP was inversely correlated with IL-6 (*r* = −0.42; *p* = 0.04), MDA (*r* = −0.46; *p* = 0.02), and positively correlated with TAC (*r* = 0.50; *p* = 0.01) (Table 4).

**Table 4:** Correlations between BAP, CTX-I, GlaOC, GluOC and markers of inflammation and oxidative stress, metabolic, and hormonal parameters in overweight/obese (Ov/Ob) and lean group of PCOS girls.

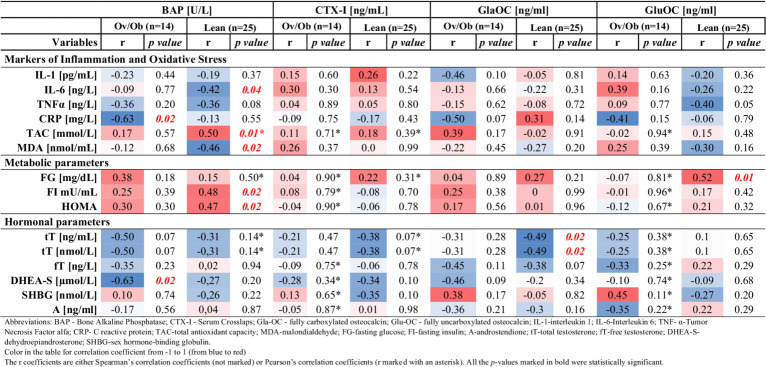

BAP, Bone Alkaline Phosphatase; CTX-I, Serum Crosslaps; Gla-OC, fully carboxylated osteocalcin; Glu-OC, fully uncarboxylated osteocalcin; IL-1, interleukin 1; IL-6, Interleukin 6; TNF-α, Tumor Necrosis Factor alfa; CRP, C reactive protein; TAC-total antioxidant capacity; MDA, malondialdehyde; FG, fasting glucose; FIfasting insulin; A, androstendione; tT, total testosterone; fT, free testosterone; DHEA-S, dehydroepiandrosterone; SHBG, sex hormone-binding globulin.

Color in the table for correlation coefficient from −1 to 1 (from blue to red).

The r coefficients are either Spearman’s correlation coefficients (not marked) or Pearson’s correlation coefficients (r marked with an asterisk). All the *p*-values marked in bold and italics were statistically significant.

Moreover, we have found significant and positive correlations in the Lean group between BAP and the metabolic parameters fasting insulin and HOMA-IR (*r* = 0.48; *p* = 0.02; *r* = 0.47; *p* = 0.02, respectively) and also between GluOC and fasting glucose (*r* = 0.52; *p* = 0.01) (Table 4).

Concerning the hormonal parameters, all significant correlations we found were negative. Namely, in the lean group, GlaOC negatively correlated with total testosterone (*r* = −0.49; *p* = 0.02), and in the Ov/Ob group, BAP negatively correlated with DHEAS (*r* = −0.63; *p* = 0.02) (Table 4).

## Discussion

PCOS in women is a complex disorder whose metabolic and hormonal characteristics, including obesity, insulin resistance, and hyperandrogenaemia, affect bone metabolism (11, 17, 31, 32).

In adult women with PCOS, BMD outcomes vary depending on body mass index (BMI). Women with PCOS and a BMI < 27 kg/m^2^ have been found to exhibit lower vertebral and nonvertebral BMD, reduced osteocalcin (bone turnover marker), and increased bone resorption marker (CTX) compared to controls. Conversely, women with PCOS and BMI ≥ 27 kg/m^2^ demonstrate higher vertebral and nonvertebral BMD without significant differences in bone turnover markers (33). Since no prospective studies are addressing these factors, it is unclear why BMD is lower in PCOS patients with a BMI < 27 kg/m^2^ compared to women without PCOS. Undoubtedly, drug therapy for PCOS is a significant confounding factor (33, 34). Some studies present conflicting evidence indicating that combined oral contraceptives (COCs), often avoided in obese and hypertensive women, may negatively impact bone mass and lower osteocalcin levels (33, 35, 36). However, the precise mechanisms through which COCs affect bone metabolism remain unclear. Another relevant medication in this context is metformin, commonly prescribed for obese or insulin-resistant PCOS patients (6). Research suggests that metformin may help preserve bone mass by reducing bone turnover, specifically by decreasing both bone formation and resorption, ultimately contributing to increased BMD (37, 38). Therefore, it can be speculated that in PCOS patients with a BMI ≥ 27 kg/m^2^, the heightened risk of vitamin D deficiency associated with metformin use could outweigh the stimulatory effects of androgens, leading to a reduction in osteocalcin levels without compromising BMD. Additionally, despite the anabolic effects of hyperinsulinemia, chronic hyperglycemia may also contribute to lower osteocalcin levels in women with a BMI ≥ 27 kg/m^2^ (39). However, in the PCOS girls participating in our study, the influence of drug therapy cannot be considered, as none of them were taking any of the aforementioned medications.

In our study in overweight and obese females, the concentrations of bone formation markers Gla-OC, Glu-OC, and bone resorption marker CTX-I were slightly lower in comparison to the matched group, but a significant difference was only observed for Gla-OC.

Increasing clinical data suggest that hyperandrogenism, hyperinsulinemia, insulin resistance, and obesity may offer in PCOS women some protective benefits for bone, while chronic low-grade inflammation and vitamin D deficiency could negatively impact bone health (40).

However, the mechanisms by which PCOS affects bone metabolism differ between adolescents and adults. During adolescence, rapid skeletal growth and hormonal changes may modulate the impact of PCOS on bone health. In adult women with PCOS, factors such as BMI, insulin resistance, hyperandrogenism, and potential pharmacological factors play a more pronounced role in determining BMD and fracture risk. These findings highlight the need for an age-specific approach when assessing and managing bone health in PCOS patients.

Unfortunately, there is a gap in the knowledge about this subject concerning the adolescent PCOS population.

Hence, our study tries to fill this research gap in this field and aims to investigate whether the endocrine, metabolic, and related inflammation and oxidative stress aberrations caused by PCOS might influence bone turnover in PCOS adolescent girls by separately considering PCOS lean and obese group of girls. This distinction is believed relevant since Ov/Ob and lean PCOS girls may experience different metabolic, endocrine, and related inflammation and OS abnormalities (10, 18) with a similar influence on the circulating levels of bone remodeling markers.

Moreover, our study aimed to determine if there is a difference between PCOS lean and overweight/obese adolescent girls (matched for chronological age and pubertal development criteria) concerning the concentration of bone turnover markers (BAP, CTX-I, and the OC forms: GlaOC and GluOC). We have observed that apart from BAP activity, the concentration of all bone markers, GlaOC, GluOC, and CTX-I, is slightly lower in the Ob/Ov group when compared to lean PCOS girls (although a significant difference was only observed for GlaOC). Aldhafiri et al. observed the same behavior of OC, but in adult PCOS [41they reported the lowest level of OC in obese PCOS, compared to slim PCOS and control subjects (41)]. Novrega da Silva also observed a reduction in bone turnover markers (OC, BAP, and CTX-I) in adolescents without PCOS but with excess weight and increased levels of glucose and insulin (in comparison to the healthy control group) (12). On the contrary, Razny proved that decreased serum level of GluOC may be a selective early symptom of insulin resistance in obese patients, and the decreased GlaOC concentration in blood is probably associated with the appearance of early markers of low-grade inflammation associated with obesity (13). Indeed, in our study, LGI marker levels were higher in the Ov/Ob group of PCOS girls than in lean females. At the same time, the Ov/Ob group manifested an increased level of metabolic markers expressed by higher concentrations of fasting glucose and insulin and higher HOMA-IR compared to lean girls.

Our results also show numerous interactions between bone markers and immune-metabolic and hormonal changes observed in both groups, Lean and Ov/Ob adolescent PCOS girls.

### Metabolic markers association with bone remodeling

In a study on non-PCOS obese children and adolescents, Garanty-Bogacka found that serum OC concentration was inversely associated with markers of the dysmetabolic phenotype, including insulin resistance, abnormal lipid profile, systemic inflammation, as well as abdominal obesity (42). Unfortunately, in our current research, we have not observed in the Ov/Ob group of girls any association between analysed metabolic markers and bone formation and resorption markers. Interestingly, in lean PCOS subjects, the circulating levels of GluOC were positively associated with fasting glucose, and BAP activity was also positively associated with fasting insulin and HOMA-IR. Pepene, et al. suggest that high circulating GluOC (a potential regulator of energy metabolism by promoting insulin production and adiponectin synthesis), may favor insulin release in lean hyperandrogenic women to compensate for impaired insulin sensitivity (31). Other authors also report that OC increases insulin sensitivity in peripheral tissues (including the bones) (11, 43–45).

### Androgens, inflammation, and redox markers association with bone remodeling

In a previous study from our group, we observed androgen abnormalities in overweight and obese girls (10). The same observation was made in the current study. Indeed, we observed significantly high concentrations of the androgens but DHEA-S value and significantly low SHBG concentration in the Ov/Ob group. Other researchers demonstrated that hormonal abnormalities observed in PCOS may be associated with a reduction in OC level in premenopausal females with BMI < 27 kg/m^2^ (11, 46).

In our study, not only the Ov/Ob group but also lean young PCOS females presented significant and negative correlations between androgens and bone markers. In the lean group, the correlation was observed between total testosterone and GlaOC (*r* = −0.49; *p* = 0.015), and in the Ov/Ob group, between DHEAS and BAP activity (*r* = −0.63; *p* = 0.0165).

On the other hand, it has been suggested that androgens may indirectly increase inflammatory markers, such as IL-1β and TNFα. Both parameters are recognized inhibitors of osteoblast differentiation and activators of osteoclastogenesis (11, 21, 47). Indeed, in our study, all inflammation markers and MDA - a marker of oxidative stress - were higher in the Ov/Ob group, and TAC was lower when compared to the Lean group. Kalyan demonstrated that chronic low-grade inflammation may negatively impact bone health in PCOS women (17). It might be expected that a similar mechanism occurs in adolescent girls. This condition may be unfavorable in this life period since this is the time of intensive growth and development, including bone tissue.

It should be highlighted that we have found many examples of negative correlations between inflammation and bone remodeling markers in both groups, such as between CRP and BAP activity in the Ov/Ob group (*r* = −0.63; *p* = 0.0156), and between IL-6 (*r* = −0.42; *p* = 0.042) and BAP activity, in the lean group. Moreover, BAP activity - bone formation marker - was also negatively correlated with the MDA - a marker of oxidative stress (*r* = −0.46; *p* = 0.022). On the other hand, BAP activity was positively correlated with TAC (*r* = 0.50; *p* = 0.012). It may suggest that low-grade inflammation, more severe in overweight and obese PCOS girls, as well as unbalanced redox homeostasis, may unfavorably influence bone formation.

### Strengths and limitations

The strength of our study is that this is the first study that presents associations between PCOS features related to PCOS pathophysiology and markers of bone health in both overweight/obese and slim adolescent girls.

The study’s main limitation is a lack of information about bone mineral density (BMD) and a large number of covariates, which may have influenced the results but were not addressed by the authors. Examples are diet and physical activity. But the comparison between the groups concerning diet and physical activity level shows that there were no significant differences between the Ov/Ob and the Lean group in macronutrients intake (including total fat, SFA, MUFA, PUFA, cholesterol, animal and plant protein, carbohydrate, fibre, and vitamins: A, D, E, K, and minerals such as Ca, Fe, Mg, and others, having a potential influence on bones), as well as in physical activity levels, expressed in MET value (unpublished results).

**Figure 2:**
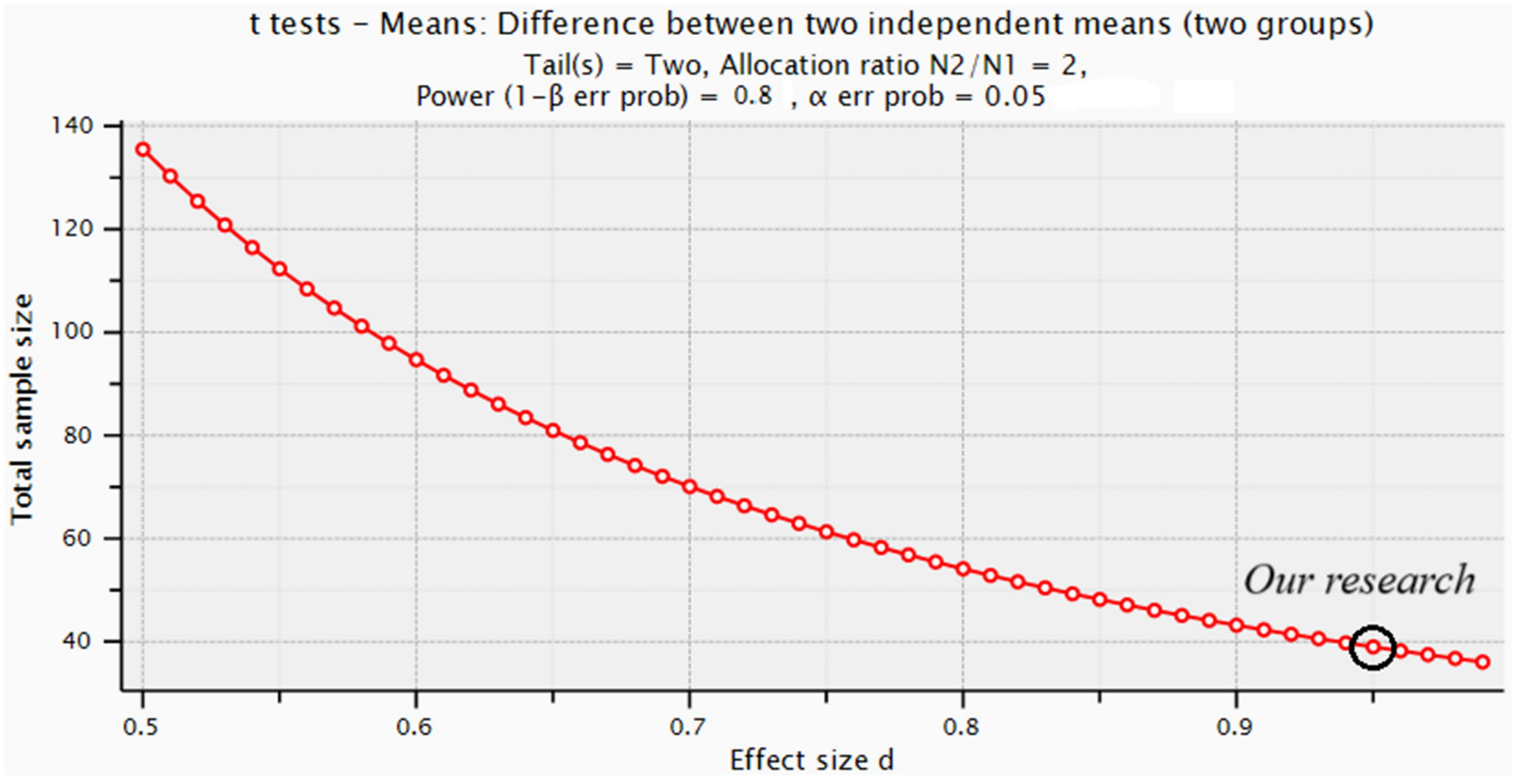
Illustrates the sample sizes required to detect smaller effect sizes (e.g., Cohen’s d = 0.5) with sufficient power.

A key limitation of this study is the relatively small sample size in the Ov/Ob group (*n* = 14), which restricts our ability to detect smaller effect sizes. As demonstrated in Figure 2, our study was only powered to detect very large effect sizes (Cohen’s d ≥ 0.95). For example, to detect medium effect sizes (Cohen’s d = 0.5), a sample size of nearly 140 participants would be required. This underscores the need for future studies with larger sample sizes to explore more subtle differences between overweight/obese and lean PCOS adolescents. Nevertheless, the significant findings reported here reflect robust, large effects that are clinically and biologically relevant.

## Conclusion

We observed that total testosterone in the lean group and DHEA-S in the Ov/Ob group of PCOS adolescents were inversely associated with bone formation markers (GlaOC and BAP activity, respectively), suggesting that these androgens may negatively influence bone remodeling. Similarly, IL-6 in the lean group and CRP in the Ov/Ob group were inversely related to BAP activity, indicating a possible detrimental effect of low-grade inflammation on bone formation, regardless of body mass.

Additionally, in the lean group, BAP activity was negatively correlated with MDA—a marker of oxidative stress—suggesting that oxidative stress may impair bone formation. Conversely, BAP activity was positively associated with TAC, and GluOC and/or BAP correlated positively with HOMA-IR, fasting glucose, and insulin, but only in lean PCOS girls. These factors may therefore support bone turnover in this subgroup.

In summary, our results suggest a potential interaction between bone markers and immune-metabolic and hormonal disturbances in both lean and Ov/Ob adolescent PCOS patients. Although further research is needed, early interventions to reduce inflammation, oxidative stress, and excess androgen levels in PCOS adolescents—regardless of weight—could help protect bone health and mineralization.

Future studies should consider including a PCOS subgroup with comorbid diabetes. In this pilot, diabetes was an exclusion criterion to reduce confounding effects, since PCOS itself increases insulin resistance and diabetes risk.

## Data Availability

The raw data supporting the conclusions of this article will be made available by the authors, without undue reservation.

## Glossary

BAP: bone alkaline phosphatase
BMI: body mass index
BMD: bone mineral density
CRP: C-reactive protein
CTX-I: C-terminal cross-linked telopeptide of type I collagen
DHEA-S: dehydroepiandrosterone sulfate
FM: fat mass
GlaOC: fully carboxylated osteocalcin
GluOC: fully uncarboxylated osteocalcin
HIP: hip circumference
HOMA-IR: homeostatic model assessment of insulin resistance
IL-1: interleukin-1
IL-6: interleukin-6
LGI: low-grade inflammation
MDA: malondialdehyde
OC: osteocalcin
OS: oxidative stress
PCOS: polycystic ovary syndrome
SHBG: sex hormone-binding globulin
TAC: total antioxidant capacity
TNFα: tumor necrosis factor-alpha
WC: waist circumference
WHR: waist-to-hip ratio
